# Prescription of Strong Opioids in Chronic Non-Cancer Pain in the Province of Valladolid (Spain)

**DOI:** 10.3390/life15010114

**Published:** 2025-01-16

**Authors:** Rodrigo Enríquez de Salamanca Gambara, Ana María Sierra Santos, Ana María Ruiz San Pedro, Federico Montero Cuadrado, Irene Muñoz León, Miguel Ángel Castro Villamor, Alicia Córdoba Romero, Ana María Del Olmo Tornero, Lucía Pérez Pérez, León Morales-Quezada

**Affiliations:** 1 Primary Care Management of Valladolid Oeste, 47012 Valladolid, Spain; 2 Primary Care Management of Valladolid Este, 47005 Valladolid, Spain; 3 Active Pain Coping Strategies Unit of Primary Care in Castile and León, 47011 Valladolid, Spain; 4 Rio Hortega University Hospital, 47012 Valladolid, Spain; 5 Advanced Clinical Simulation Center, Faculty of Medicine, Universidad de Valladolid, 47002 Valladolid, Spain; 6 Medical Team Coordination Unit, Primary Care Management of Valladolid Oeste, 47012 Valladolid, Spain; 7 Medical Team Coordination Unit, Primary Care Management of Valladolid Este, 47005 Valladolid, Spain; 8 Spaulding Rehabilitation Hospital, Department of Physical Medicine & Rehabilitation, Harvard Medical School, Boston, MA 02115, USA

**Keywords:** opioids, non-cancer chronic pain, primary care

## Abstract

**Background:** Chronic non-cancer pain (CNCP) is one of the leading causes of disability. The use of strong opioids (SOs) in the management of CNCP is increasing, although evidence supporting their use remains limited. Primary care (PC) plays a key role in this context. **Objectives:** Our objectives were to determine the prevalence and profile of patients using SOs for CNCP in PC consultations in Valladolid in 2022, and to describe the consumption of SO prescribed for CNCP from 2020 to 2023. **Methods:** We conducted a descriptive and retrospective study using data extracted from the Pharmaceutical Consumption Information System of Castilla y León. Patients in Valladolid with SO use for more than three months due to CNCP were analyzed. Sociodemographic and clinical characteristics of these patients in 2022 were described. The number of defined daily doses (nº DDDs) and costs from 2020 to 2023 were analyzed. **Results:** A total of 3642 patients were included (0.7% of the population of Valladolid), 71.8% of whom were women. Of the patients, 62.4% were aged 70 or older, 39.8% lived in rural areas, and 9.9% resided in nursing homes. The most frequently prescribed SOs in nº DDDs were fentanyl and tapentadol. The highest consumption in nº DDDs was in patients who lived in nursing homes, were over 70 years old and were resident in rural areas. The number of DDDs from 2020 to 2023 for SOs in DCNO increased by 41%. **Conclusions:** In total, 0.7% of the population of Valladolid consumes SOs for CNCP, mostly women and people over 70 years old. The consumption of strong opioids in DDDs grew by 41% from 2020 to 2023.

## 1. Introduction

The International Association for the Study of Pain (IASP) describes pain as “an unpleasant sensory and emotional experience associated with, or resembling that associated with, actual or potential tissue damage.” Pain can be acute or chronic and is a multifactorial condition with biological, psychological, and social components [1,2,3].

Chronic non-cancer pain (CNCP) is defined as pain that persists for more than three months and is not associated with cancer processes. Among the conditions included in CNCP, lower back pain and neck pain stand out due to their high prevalence and because they are among the leading causes of years lived with disability [4]. The prevalence of CNCP is estimated to be 20% in the United States and Europe, and 25% in Spain [5,6,7].

Pain has traditionally been treated with medications based on the World Health Organization’s (WHO) analgesic ladder. This tool, introduced in 1986, was originally intended to promote the use of opioids for cancer pain management but has since been applied to CNCP as well. Opioids are classified by their potency into weak opioids (e.g., tramadol and codeine) and strong opioids (SOs) (e.g., morphine, fentanyl, hydromorphone, oxycodone, tapentadol, and buprenorphine) [8,9].

The use of opioids for CNCP is increasing annually [10], with more potent opioids being prescribed more frequently, for longer durations, and at higher doses [11,12].

However, the benefits of opioid use in CNCP in terms of functionality and quality of life improvements remain unproven [13,14,15,16,17]. Prolonged opioid use can lead to tolerance, reducing effective pain relief, and carries risks such as opioid-induced hyperalgesia, endocrine hypogonadism, drowsiness, dependence, opioid use disorder, sleep apnea, falls, overdose, and death [16,18]. These risks are amplified when opioids are co-prescribed with other medications such as benzodiazepines, antidepressants, or gabapentinoids, especially in older patients [17].

The rising consumption of opioids for CNCP is a concern in many countries [19,20]. For instance, in Spain, the Interterritorial Council of the National Health System has approved the Optimization Plan for Opioid Analgesic Use in CNCP. This plan aims to optimize prescribing practices, strengthen pharmacotherapeutic monitoring, enhance addiction management, improve communication with patients, raise public awareness, and monitor opioid consumption [21].

Primary care (PC) plays a key role in addressing this issue, as pain is the most common reason for consultation (60%). Chronic pain accounts for more than 50% of pain-related visits in PC [7]. Both PC physicians and hospital-based physicians can prescribe these medications, but it is primarily the PC physician who monitors the treatment, making primary care a critical point for the control of opioid use [22].

Main Objective: The primary objective of this study is to determine the prevalence of strong opioid (SO) use for chronic non-cancer pain (CNCP) in primary care (PC) consultations in the province of Valladolid and to describe the profile of patients undergoing treatment in 2022. Secondary Objectives: Additionally, we aim to describe the consumption and cost of SO prescribed for CNCP from 2020 to 2023.

## 2. Methods

A descriptive and retrospective study was conducted in the PC setting of the province of Valladolid (Spain) on patients treated with SO for more than three months due to non-cancer health issues.

Morphine, oxycodone, oxycodone/naloxone, hydromorphone, fentanyl, tapentadol, and buprenorphine were considered strong opioids according to the WHO analgesic ladder classification [8,9].

The source of data was the Castilla y León Pharmaceutical Consumption Information System (CONCYLIA), which contains information on dispensations through prescriptions from the public national health system (SNS) at pharmacies in Castilla y León, Valladolid, Spain. Data extraction was performed by the primary care pharmacy services of the SNS in Valladolid.

First, the use of these drugs and the profile of patients consuming them were analyzed for the year 2022. Subsequently, the consumption and expenditure trends for SOs were studied from 2020 to 2023, following methods used in similar studies [12]. The patient profile study was limited to a specific year, in this case 2022, due to the manual extraction required for clinical data from the CONCYLIA program. This limitation was necessary as 2022 was the first year with complete data available when the research project began.

Study Population: The study included all individuals with a national health system individual health card in the Valladolid health areas (East and West). According to the Spanish National Statistics Institute, approximately 517,975 people resided in the province of Valladolid in 2022 [23]. Inclusion Criteria: Individuals aged ≥18 years with a prescription for SO by National Health System physicians for more than three months for CNCP between 2020 and 2023. Exclusion Criteria: Patients treated for three months or less, those with a cancer diagnosis, and/or those receiving parenteral opioids to ensure that SO prescriptions were not associated with cancer pain management.

Variables: In 2022, the following variables were analyzed: age, sex, place of residence (rural or urban), and whether the patient resided in a nursing home. Diagnoses associated with SO prescriptions were categorized according to clinical practice guidelines and the Global Burden of Diseases classifications [4,17] into musculoskeletal (MSK) pain related to the spine, MSK pain unrelated to the spine, non-MSK pain, and neuropathic pain. The active ingredient of the prescribed SO was classified based on the Anatomical Therapeutic Chemical (ATC) classification system, and routes of administration (nasal, oral, sublingual, and transdermal) were noted, along with the number of dispensed packages, defined daily doses (DDDs), annual cost, and the number of different SO active ingredients. The consumption of other analgesics or adjuvant medications during the same year was also analyzed: nonsteroidal anti-inflammatory drugs (NSAIDs), benzodiazepines, gabapentinoids, muscle relaxants, antidepressants, antipsychotics, weak opioids, and local anesthetics. The DDD calculation formula is detailed in Supplement S1.

Statistics and Data Analysis: All data were stored in an Excel database after data cleansing to identify outliers and duplicates. Statistical analysis was performed using the SPSS 25.00 software package (SPSS Inc^®^, Chicago, IL, USA). A descriptive analysis of the sample was conducted. Qualitative variables were summarized with their frequency distribution, and quantitative variables with their mean and standard deviation (SD). The distribution of variables was assessed against theoretical models using the Kolmogorov–Smirnov and Levene tests. In cases of variance asymmetry, the median and interquartile range (IQR) were calculated, and non-parametric tests were used for variable comparisons. For comparisons of quantitative variables, Student’s *t*-test was used for normally distributed data (when there was equality of variances), and the Mann–Whitney U test for non-normal distributions. The chi-square test was employed for 2 × 2 contingency tables or proportion contrasts to establish associations or dependencies between qualitative variables, and Fisher’s exact test was applied when more than 25% of expected frequencies were less than 5. A 95% confidence level (*p* < 0.05) was considered statistically significant for all tests.

Ethical Considerations: This study was approved by the Ethics and Research Committee for Medicinal Products of the Valladolid East and West health areas under reference codes 23-Pl128 and PI-GR23-3285. The research adhered to the Code of Good Scientific Practice and the legal framework composed of the following regulations: Organic Law 3/2018 of December 5 on the Protection of Personal Data and Guarantee of Digital Rights, Law 14/2007 of July 3 on Biomedical Research, and Law 14/2011 of June 1 on Science, Technology, and Innovation. The project was conducted with strict respect for patient confidentiality (anonymized data). The researchers committed to following the principles and recommendations of the Declaration of Helsinki for biomedical research involving humans, including studies on human material and data.

## 3. Results

Of the 3764 patients who met the study’s inclusion criteria, 122 were excluded due to diagnostic coding errors.

In 2022, a total of 3642 patients were treated with one or more strong opioids for more than three months due to a non-cancer condition, representing 0.7% of the population in Valladolid. The median age was 75 years (IQR 62–84). Of these, 62.4% were aged 70 or older, and 37.9% were aged 80 or older. Women comprised 71.8% of the cohort, and 9.9% of the patients lived in nursing homes. Additionally, 39.8% resided in rural health zones. A sex-based comparison is shown in Table 1.

A total of 81.1% of patients had a diagnosis associated with musculoskeletal pain, with 50.6% related to the spine and 31.4% unrelated to the spine. Another 14.6% had a diagnosis associated with non-musculoskeletal pain, and 3.3% had neuropathic pain (see Supplement S1, Table S1).

Tapentadol was used by 42% of patients, fentanyl by 27.5%, and oxycodone-naloxone by 23.2%. In nursing homes, 41.2% of patients were treated with fentanyl, 25.1% with tapentadol, 20.1% with oxycodone-naloxone, 15% with buprenorphine, 1.4% with morphine, 1.4% with hydromorphone, and 0.6% with oxycodone.

The most used administration route was oral, in 2404 patients (66%), followed by transdermal in 1232 patients (33.8%). A sex-based comparison is shown in Table 2. Among transdermal administered drugs, 73.2% of prescriptions were for fentanyl (911 patients).

A total of 224 patients were prescribed two different strong opioid (SO) active ingredients, 25 patients were prescribed three, and 1 patient was prescribed four. The median age of these patients was 74 years (IQR 61–82). Among them, 74.4% were women, and 6.4% resided in nursing homes.

In the analysis of consumption by active ingredient in 2022, the highest number of defined daily doses (DDDs) dispensed was for fentanyl, followed by tapentadol (251,736 and 184,151 DDD, respectively). However, tapentadol accounted for the highest expenditure, representing 46% of the total cost (see Table 3).

When analyzing the number of DDDs by sex, age, rurality, and residence in nursing homes, statistically significant differences were observed for age, rurality, and residence in care facilities but not for sex (see Table 4).

In the analysis of other treatments prescribed that same year, 61.2% of patients had a prescription for benzodiazepines, 58.2% for weak opioids, 50.3% for antidepressants, 50% for NSAIDs, 42.6% for gabapentinoids, 16.1% for antipsychotics, and 3% for muscle relaxants (see Table 5).

An analysis of the trend from 2020 to 2023 shows that opioid consumption in Valladolid increased by 41.50% since 2020, rising from 506,928 DDD in 2020 to 717,290 DDD in 2023, with a progressive increase each year (see Table 6). While fentanyl predominated in the total DDD, oxycodone showed the highest percentage increase, with a rise of 155% (see Table 6). Expenditure increased by 21.70% compared to 2020 (see Supplement S1).

## 4. Discussion

This study analyzes the prevalence and patient profile of those treated with SOs for CNCP in the province of Valladolid in 2022, as well as the evolution of opioid consumption in CNCP from 2020 to 2023.

In the analysis of the evolution of opioid consumption for CNCP, we observe an increase in the number of defined daily doses (DDDs) and overall spending. This finding aligns with global opioid consumption data from the Spanish government and similar studies conducted on CNCP in other autonomous communities in Spain [12,24,25].

Comparisons with other provinces and time periods proved challenging. Studies on SO use for CNCP employ highly varied methodologies. SO classifications differ between studies; there are studies that include some and others all opioids (strong and weak), while others focus solely on new prescriptions, or on patients consuming high doses of morphine milligram equivalents. Additionally, data sources vary significantly between countries due to differing healthcare models [12,19,20,26,27,28]. Consequently, comparing prevalence across regions is complex, although all studies indicate an increase in opioid prescriptions for CNCP [12,19,20,26,27,28]. We were able to compare our findings with the study by A. Perelló Bratescu et al., titled “Trends in the Prescription of Strong Opioids for Chronic Non-Cancer Pain in Primary Care in Catalonia: Opicat-Padris-Project” becouse our methods were similar [12]. The percentage of the population in Valladolid represented by our study is 0.7%, a high figure compared to the 0.29% in Aina Perelló-Bratescu’s study conducted in Catalonia in 2017. Considering that the percentage of individuals over 65 years old in Catalonia in 2017 was 19% and in Valladolid in 2022 was 24%, and knowing that opioids are increasingly prescribed more frequently to older individuals, this could explain the difference [23,26]. No other studies were found that isolate the percentage of the population consuming only SO for CNCP, making this a revealing finding [27,28].

The median age in our study was comparable to that of other studies [12]. The data reveal that the highest percentage of opioid prescriptions were given to individuals over 80 years old. Additionally, a higher number of DDDs were dispensed to patients over 70 years old than to younger patients. The use of opioids in CNCP has not demonstrated benefits in managing musculoskeletal pain in the elderly [26,29]; furthermore, the emergence of side effects such as overdose risk, increased falls, altered consciousness, and mortality is more common in older adults [30].

In Valladolid, it is estimated that around 7001 patients reside in a nursing home, which represents 1.35% of the province’s population [31]. The number of patients treated with opioids in these facilities is 359, meaning 5.1% of the patients living in long-term care centers in Valladolid were treated with opioids. This is a very high percentage compared to the general population’s opioid consumption in Valladolid (0.7%). Unlike others, the most consumed active ingredient in these patients was fentanyl, and the number of DDDs consumed was significantly higher. Some studies indicate that opioid use in long-term care centers has doubled in the past decade, and concurrent use with interacting drugs is common, which should make us reflect on whether we are practicing medicine appropriately [32,33,34].

Regarding sex, it is common in many studies related to opioid prescriptions and pain that there is a higher percentage of women, as in our study [35,36]. Gender-related factors that increase opioid consumption in CNCP include having rheumatoid diseases, fibromyalgia, smoking, poor self-perception of health, and a greater tendency of doctors to prescribe analgesics to women, among others. Biopsychosocial mechanisms may be behind these inequalities [37,38,39,40,41,42]. As for the DDDs by sex, no significant differences were found. Helena de Sola’s systematic review of factors associated with general opioid consumption indicated that factors such as being male, young, and having chronic pain were related to opioid use [27]. Therefore, it seems that opioid consumption in CNCP has particularities in its distribution by sex.

In the province of Valladolid in 2022, 58% of the population lived in urban areas, a figure very similar to that of our study (60%) [24,43]. However, when analyzing the DDDs between rural and urban patients separately, we observed an increase in DDDs among rural patients. There is controversy over whether rurality is a risk factor for opioid consumption, due to factors such as greater difficulty accessing non-pharmacological resources for pain management [12,24,43]. In our study, rurality does appear to be a determinant.

A wide range of diagnoses associated with opioid prescriptions was analyzed, and a high prevalence of musculoskeletal and non-musculoskeletal pain was observed, despite clinical practice guidelines not recommending opioids for these indications [17]. Prescriptions for neuropathic pain were also found, although guidelines recommend them as a third-line treatment [44]. Additionally, for some diagnoses associated with opioid prescriptions, such as fibromyalgia, complex multimorbidity, and mental health issues, opioids are contraindicated [17].

Tapentadol and fentanyl were the most prescribed drugs in the study, despite clinical guidelines recommending morphine as the first choice [45]. Although the number of patients treated with tapentadol in our study was higher, the number of DDDs for fentanyl was greater. Tapentadol was the drug with the highest expenditure in all years of the study, despite having fewer DDDs than fentanyl due to its higher price [46]. The reasons behind these prescribing trends for more expensive drugs should be investigated [47].

Problematic opioid use seems to be common among patients with chronic pain: nearly one in ten experience opioid dependence or abuse disorder, one in three shows signs or symptoms of dependence or abuse disorder, and one in five exhibits aberrant behavior [48]. Factors such as consuming high opioid doses, long durations of use, conditions like fibromyalgia, substance abuse history, age, or having many different doctors are risk factors for improper opioid use [20,28]. It would be interesting to analyze these factors in our study and their relationship with the number of DDDs per patient.

Regarding the prescription of other medications during 2022, noteworthy figures of minor opioids, benzodiazepines, antidepressants, and gabapentinoids were observed. These figures were somewhat contradictory when compared to similar studies [33,49,50]. While our study cannot correlate the simultaneous use of these medications with opioids, it should be noted that benzodiazepines combined with opioids are contraindicated due to the risk of respiratory depression and increased mortality [51,52]. Antidepressants, when used alongside opioids, increase the risk of serotonin syndrome, especially with tapentadol [25,53], and when combined with gabapentinoids, increase the risk of mortality [51,54].

The increase in opioid consumption for CNCP, associated with factors such as gender, age, and place of residence, may be due to various reasons: the difficult access to non-pharmacological alternatives for pain treatment, the demand for immediate pain relief, high expectations for drug effectiveness, delays in specialized consultations, lack of training, and the great complexity primary care professionals face in managing pain and suffering daily [22,55,56].

In a recent study on health priority setting in Australia, the deprescribing of opioids in primary care has been identified as a top research priority [57]. There are already some experiences with opioid deprescribing in countries like the United Kingdom, which have resulted in reduced opioid use and improved pain interference with daily activities compared to usual care [58]. Deprescribing has proven to be a key clinical strategy to reduce opioid-related harm and improve quality of life, although individuals prescribed high doses of opioids appear less likely to complete gradual reduction [59,60].

Analyzing these data is a crucial first step to understand the situation in Valladolid. However, we believe it would be beneficial to undertake future national, European, and international projects to understand the situation in other territories. In Spain, the Interterritorial Council of the National Health System has approved the ’Plan for Optimizing the Use of Opioid Analgesics in CNCP’ [21]. Our data will be shared with public health services to reach as many healthcare professionals, patients, and the general population as possible, raising awareness of this situation and implementing measures to optimize opioid prescriptions in CNCP in line with the national plan.

## 5. Conclusions

The consumption of DDD increased by 41% from 2020 to 2023. Tapentadol accounted for the highest expenditure in all years studied. In 2022, 0.7% of Valladolid’s population used SO for CNCP, most of whom were women and over 70 years old. Differences in DDDs dispensed were observed based on patient age, urban or rural residence, and whether they lived in nursing homes. Benzodiazepines, weak opioids, gabapentinoids, and antidepressants were frequently prescribed to these patients in 2022. These findings are critical for implementing measures to adjust the consumption of these drugs in our territory.

## 6. Limitations

Our study is based on the analysis of patient characteristics consuming SOs over the year 2022 and trends from 2020 to 2023; however, it is not without limitations. There is controversy regarding whether tapentadol is a high- or moderate-potency opioid due to its lower affinity for μ-opioid receptors. However, the WHO includes it in the third step of strong opioids, and in line with other similar studies, we decided to include it [61,62,63].

Additionally, this study covers the years of the SARS-CoV-2 pandemic, which may have influenced the consumption of analgesic medications for CNCP, as non-pharmacological pain management resources were less available due to the saturation of the Spanish national health system [64].

For this reason, when analyzing patients who consumed more than one SO or adjuvant medications during that year, we cannot determine if the consumption was simultaneous. The 253 patients treated with two or more SO in 2022 were analyzed separately. No differences in age or sex were found in the initial analysis of this group, but further investigation is warranted to assess whether a portion of the population was consuming multiple SO simultaneously or an SO alongside a weak opioid, posing health risks [17].

Furthermore, this study is based on pharmacy dispensing data, so the actual consumption of medications cannot be confirmed, and our conclusions are putative. Adherence to these treatments may be irregular, so our study is an approximation of the reality of opioid consumption in Valladolid. In addition, it is a retrospective study, which limits the validity of the data analysis compared to a prospective study. Some prescriptions had non-specific diagnoses, which complicates categorization, and prescriptions without a diagnosis were excluded, which could introduce a margin of error in prevalence estimates. Opioids prescribed in hospitals or drug treatment centers were also not included. Chronic SO dispensations for CNCP from systems outside the national health system were excluded, although Spain’s public health system is the primary provider for chronic medication dispensations.

## Figures and Tables

**Table 1:** Baseline characteristics of the study population in 2022.

Variable	N	N Male	N Female	*p*-Value ^b^
Sex ^a^	3642	1027 (28.2%)	2615 (71.8%)	<0.01
Age				<0.01
18–29 years	1 (0.02%)	0 (0%)	1 (0.03%)	
30–39 years	42 (1.2%)	19 (1.9%)	23 (0.9%)	
40–49 years	212 (5.8%)	103 (10%)	109 (4.2)	
50–59 years	464 (12.7%)	198 (19.3%)	266 (10.2%)	
60–69 years	654 (18%)	229 (22.3%)	425 (16.3%)	
70–79 years	892 (24.5%)	238 (23.2%)	654 (25%)	
80–89 years	998 (27.5%)	181 (17.6%)	817 (31.2%)	
>90 years	379 (10.4%)	59 (5.7%)	320 (12.2%)	
Geographic distribution				<0.01
Urban	2190 (60.2%)	579 (56.4%)	1611 (61.6%)	
Rural	1454 (39.8%)	448 (43.6%)	1004 (38.4%)	
Nursing home				<0.001
Yes	359 (9.9%)	68 (6.6%)	291 (11.1%)	
No	3283 (90.1%)	959 (93.4%)	2324 (88.9%)	

^a^ Values expressed as total numbers (percentage) or medians (25th–75th percentile), as appropriate. ^b^ The Mann–Whitney U test or chi-squared test was used, as appropriate. N: Number of patients in the study.

**Table 2:** Strong opioids and administration route compared by sex in 2022.

Drug	N (Porcentaje)	Male	Female	*p*-Value ^b^
Sex ^a^	3642	1027 (28.2%)	2615 (71.8%)	<0.01
Morphine	94 (2.6%)	19 (2%)	32 (1.2%)	
Hydromorphone	63 (1.7%)	11 (1.1%)	35 (1.4%)	
Oxycodone	38 (1%)	10 (1%)	14 (0.6%)	
Oxycodone-naloxone	844 (23.2%)	223 (22.9%)	504 (20.4%)	
Fentanyl	1003 (27.5%)	183 (18.8%)	661 (26.8%)	
Buprenorphine	349 (9.6%)	69 (7.1%)	268 (10.9%)	
Tapentadol	1528 (42%)	458 (47.1%)	954 (38.7%)	
Administration route				<0.01
Nasal	1 (0.02%)	1 (0.1%)	0 (0%)	
Oral	2404 (66%)	1650 (63.1%)	754 (73.4%)	
Sublingual	3 (0.1%)	2 (0.1%)	1 (0.1%)	
Transdermal	1232 (33.8%)	960 (36.7%)	272 (26.5%)	

^a^ Values expressed as total numbers (percentage) or medians (25th–75th percentile), as appropriate. ^b^ The Mann–Whitney U test or chi-squared test was used, as appropriate. N: number of patients in the study.

**Table 3:** Number of DDDs, drug packaging, billed amount per active substance in 2022.

Drug	N.º DDDs	Drug Packaging	Billed Amount (EUR)
Morphine	12,760	1780	19,502
Hydromorphone	8778	904	23,685
Oxycodone	6784	943	13,833
Oxycodone-naloxone	104,332	10,696	354,665
Fentanyl	251,736	22,360	511,762
Buprenorphine	92,930	4714	142,969
Tapentadol	157,080	16,658	909,174
TOTAL	634,401	58,055	1,975,590

**Table 4:** Comparison of the baseline characteristics of the study population based on DDD in 2022.

Category Analyzed	Median DDD (IQR)	*p*-Value ^b^
Variable ^a^		0.504
Sex	112 (62–225)	
Famale	105 (60–216)	
Male		0.027
Age	105 (56–217)	
>70 years	119 (67–225)	
<69 years		0.008
Geographic distribution	119 (67–240)	
Rural	106 (60–210)	
Urban		0.001
Nursing home	109 (60–217)	
No nursin home	134 (71–270)	

DDD: Defined daily dose. ^a^ Values expressed as total numbers (percentage) or medians (25th–75th percentile), as appropriate. ^b^ The Mann–Whitney U test or chi-squared test was used, as appropriate.

**Table 5:** Other drugs prescribed in the year 2022.

Drug	N
NSAIDs	1812 (50%)
Benzodiazepines	2221 (61.2%)
Gabapentinoids	1544 (42.6%)
Muscle relaxants	109 (3%)
Antidepressants	1824 (50.3%)
Antipsychotics	586 (16.1%)
Local anesthetics	277 (7.6%)
Weak opioids	2109 (58.2%)

N: Number of patients in the study. NSAID: Nonsteroidal anti-inflammatory drug.

**Table 6:** Trend from 2020 to 2023 shows that opioid consumption according to the nº DDDs.

	2020	2021	2022	2023	Increase 2020–2023
Morphine	7166	9509	12,759	15,031	109.75%
Hydromorphone	6900	9030	8778	6630	−3.91%
Oxycodone	2993	5034	6784	7637	155.18%
Oxycodone-naloxone	97,521	101,147	104,331	109,565	12.35%
Fentanyl	193,674	235,913	251,736	304,032	56.98%
Buprenorphine	79,625	88,244	92,930	90,242	13.33%
Tapentadol	119,047	136,653	157,080	184,151	54.69%
Total	506.928	585.532	634.400	717.290	41.50%

## Data Availability

Data are available upon request.

## Supplementary Materials

The following supporting information can be downloaded at: https://www.mdpi.com/article/10.3390/life15010114/s1. Index: 1. Number of DDDs. 2. Diagnoses associated with medication. 3. Medication expenditure: economic cost.
