# Supplementary material for: Prescription of Strong Opioids in Chronic Non-Cancer Pain in the Province of Valladolid (Spain)
Source: Life (Basel). 2025 Jan 16;15(1):114. doi: 10.3390/life15010114 (PMC11766594; doi:10.3390/life15010114)
Supplement: Supplementary file 1 [file life-15-00114-s001.zip › life-3372490-supplementary.pdf]

life MDPI

Supplement 1:

Index:

1. **Number of DDDs**
2. **Diagnoses associated with medication:**
   a. **Table 1: Musculoskeletal Pain**
   b. **Table 2: Neuropathic Pain**
   c. **Table 3: Non-Musculoskeletal Pain**
3. **Medication expenditure: economic cost**

1. **Formula to calculate the number of DDDs:**

(Number of packages dispensed annually for each pharmaceutical specialty × number of dosage forms per package × milligrams of active ingredient per dosage form) / DDD in milligrams.

2. **Diagnoses associated with medication**

Table S1. Musculoskeletal Pain

Diagnosis Related to the Spine	Number of Patients (Percentage)
Low back pain	354 (9.6%)
Spinal canal stenosis	272 (7.4%)
Lumbar sciatica	237 (6.5%)
Lumbar herniated disc	228 (6.2%)
Lumbar osteoarthritis	143 (3.9%)
Vertebral compression fracture	138 (3.7%)
Herniated disc	117 (3.2%)
Cervical brachialgia	83 (2.2%)
Vertebral fracture	56 (1.5%)
Other spinal problems	39 (1%)
Scoliosis	34 (0.9%)
Dorsalgia	32 (0.8%)
Facet syndrome	32 (0.8%)
Spinal fusion surgery	30 (0.8%)
Cervical osteoarthritis	13 (0.3%)
Unspecified back pain	12 (0.3%)
Spondylolisthesis	10 (0.3%)
Cervical pain	8 (0.2%)
Sacral pain	5 (0.1%)
Total	1,843 (50.5%)
**Diagnosis: non related to the spine**	
Non-Specified Musculoskeletal Pain	191 (5.2%)
Osteoarthritis (Unspecified)	168 (4.6%)
Non-Specified Chronic Pain	107 (2.9%)
Rheumatic Disease	100 (2.7%)
Knee Osteoarthritis	95 (2.6%)

Hip Osteoarthritis	85 (2.3%)
Coxalgia	64 (1.8%)
Gonalgia	64 (1.8%)
Fibromyalgia	45 (1.2%)
Prosthesis Carrier	44 (1.2%)
Omalgia	35 (1%)
Osteoporosis	26 (0.7%)
Lower Limb Pain	24 (0.7%)
Trauma	17 (0.5%)
Non-Specified Generalized Pain	16 (0.4%)
Osteoarthritis of Lower Extremities	13 (0.4%)
Thoracic Wall Pain	9 (0.2%)
Upper Limb Pain	8 (0.2%)
Hip Fracture	8 (0.2%)
Upper Limb Fracture	7 (0.2%)
Osteoarthritis of Upper Extremities	5 (0.1%)
Fractures	4 (0.1%)
Chronic Pain Syndrome	4 (0.1%)
Myofascial Syndrome	4 (0.1%)
Lower Limb Fracture	3 (0.1%)
Total	1146 (31.4%)

Table S2: Neuropathic Pain

**Diagnosis**	**Number of Patients (Percentage)**
Neuropathic pain	69 (1.9%)
Postherpetic neuralgia	20 (0.5%)
Radiculopathy	19 (0.5%)
Trigeminal neuralgia	8 (0.2%)
Unspecified neuralgia	5 (0.1%)
Total	121 (3.3%)

Table S3: Non-Musculoskeletal Pain

Diagnosis	**Number of patients (percentage)**
Others	348 (9.5%)
Complex Multimorbidity	44 (1.2%)
Neurological Disease	26 (0.7%)
Complex Regional Pain Syndrome	12 (0.3%)
Amputation	9 (0.2%)
Peripheral Arterial Disease	9 (0.2%)
Gastrointestinal Issues	9 (0.2%)
Headache	8 (0.2%)
Unspecified Pain	8 (0.2%)
Pulmonary Disease	8 (0.2%)

Mental Health Issues	8 (0.2%)
Skin Ulcer	7 (0.2%)
Cardiac Disease	6 (0.2%)
Neuropathy	6 (0.2%)
Gastrointestinal Problems	6 (0.2%)
Pelvic Pain	5 (0.1%)
Myelopathy	5 (0.1%)
Unspecified Pain	4 (0.1%)
Urological Symptoms	3 (0.1%)
**Total**	**531 (14.6%)**

3. **Medication expenditure: economic cost**

	**2020**	**2021**	**2022**	**2023**	**incremento 2023/2020**
Morphine	10812,1	14473,2	19501,85	22941,07	112,18%
Hydromorphone	18617,56	24364,35	23684,83	18515,18	-0,55%
Oxycodone	6104,23	10267,41	13833,47	15556,23	154,84%
Oxycodone-naloxone	331517,87	343840,31	354664,89	372459,14	12,35%
Fentanyl	452344,65	613874,32	511762,15	612363,16	35,38%
Buprenorphine	122554,75	135919,79	142968,92	138725,86	13,20%
Tapentadol	685360,26	785018,61	909173,94	799801,24	16,70%
**TOTAL**	1627311,42	1927757,99	1975590,05	1980361,88	21,70%
